# Endonephrology: Evolving Techniques of Endoscopic Ultrasound in Diagnosing Renal Lymphoma

**DOI:** 10.14309/crj.0000000000001815

**Published:** 2025-08-13

**Authors:** Erika Tsuchiyose, Michael Talanian, Erik Holzwanger

**Affiliations:** 1 Tufts University School of Medicine, Boston, MA; 2 Center for Advanced Endoscopy, Division of Gastroenterology, Tufts Medical Center, Boston, MA

**Keywords:** endoscopic ultrasound, EUS-guided fine needle biopsy, MALT lymphoma, lymphoma

## Abstract

Mucosa-associated lymphoid tissue lymphoma is a subtype of non-Hodgkin lymphoma, exceedingly rare when originating in the kidney. Most renal masses are percutaneously sampled with ultrasound or computed tomography guidance; however, limitations can include location, size, and body habitus, which may be resolved using endoscopic ultrasound–guided approaches. We present an unusual case of a 77-year-old man with history of systemic lupus erythematosus, duodenal mucosa–associated lymphoid tissue lymphoma in remission, and rectal adenocarcinoma who presented for evaluation of a right renal mass, which highlights the novelty of endoscopic ultrasound–guided renal evaluation and tissue acquisition.

## INTRODUCTION

Mucosa-associated lymphoid tissue (MALT) lymphoma is a subtype of non-Hodgkin lymphoma most commonly of gastric origin, accounting for approximately 7%-8% of newly diagnosed lymphomas each year.^1,2^ The lungs, salivary glands, and ocular adnexa are other common sites of origin,^3^ but the duodenum is an infrequent primary site for MALT lymphoma.^4^ Although typically a localized disease, MALT lymphoma can metastasize to regions such as the lymph nodes and bone marrow; however, renal involvement remains highly unusual.^5^ Renal biopsies are traditionally gathered percutaneously with ultrasound or computed tomography (CT) guidance. Minimally invasive endoscopic ultrasound (EUS)-guided approaches are infrequently reported in the literature.^6^ We present a novel case of EUS-guided fine needle biopsy (FNB) used to diagnose renal MALT lymphoma.

## CASE REPORT

**Figure 1.**
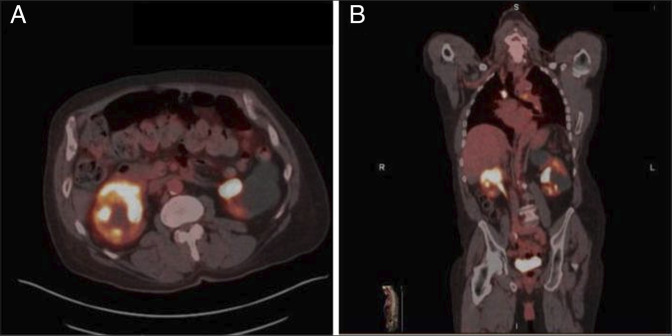
(A) Axial MRI highlighting poorly circumscribed right renal mass with intense FDG uptake. (B) Sagittal MRI demonstrating renal mass and mediastinal lymphadenopathy with FDG uptake. FDG, fluorodeoxyglucose; MRI, magnetic resonance imaging.

A 77-year-old African American man with a history of duodenal MALT lymphoma with multiple recurrences, prostate cancer, newly diagnosed rectal adenocarcinoma, and systemic lupus erythematosus (SLE) presented for evaluation of a right renal mass seen on prior imaging. Duodenal MALT lymphoma was diagnosed in 2007 and achieved complete remission with cyclophosphamide, vincristine, and prednisone. Recurrences in 2008 treated with rituximab, 2010 with cyclophosphamide, vincristine, and prednisone, and 2015 with rituximab/bendamustine therapy all achieved interval complete remission. In 2024, surveillance CT scan revealed an ill-defined, enhancing lesion in the superior pole of the right kidney. Subsequent positron emission tomography imaging demonstrated a poorly circumscribed 5.8-cm mass at the mid-pole of the right kidney with intense fluorodeoxyglucose uptake (Figure 1). There was also intensely fluorodeoxyglucose-avid multifocal mediastinal and bilateral hilar lymphadenopathy.

**Figure 2.**
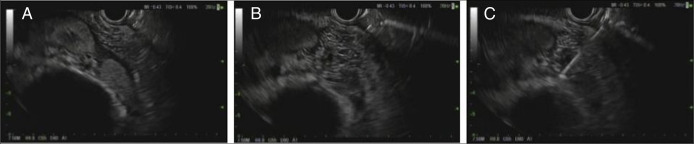
(A) Endoscopic ultrasound demonstrating right renal parenchyma. (B) Irregular, right renal mass. (C) Renal mass fine needle biopsy.

Multidisciplinary discussion with medical oncology and interventional radiology deemed percutaneous sampling challenging because of the location of the mass, so endoscopy was performed and revealed no evidence of luminal disease. Subsequent EUS identified a hypoechoic, heterogeneous mass in the right kidney measuring 35 mm in maximal cross-sectional diameter (Figure 2). Endosonographic borders were poorly defined, but the mass abutted the duodenal wall. Color Doppler imaging was used before needle puncture to confirm lack of significant vascular structures within the needle path and FNB was performed. Three passes were made with a 22-gauge biopsy needle using a trans-gastric and trans-duodenal approach (Figure 2). Lymph nodes could not be visualized on EUS. The differential diagnosis included MALT lymphoma, metastatic rectal adenocarcinoma, and exceedingly unlikely, granulomatous disorders such as tuberculosis or sarcoidosis.

**Figure 3.**
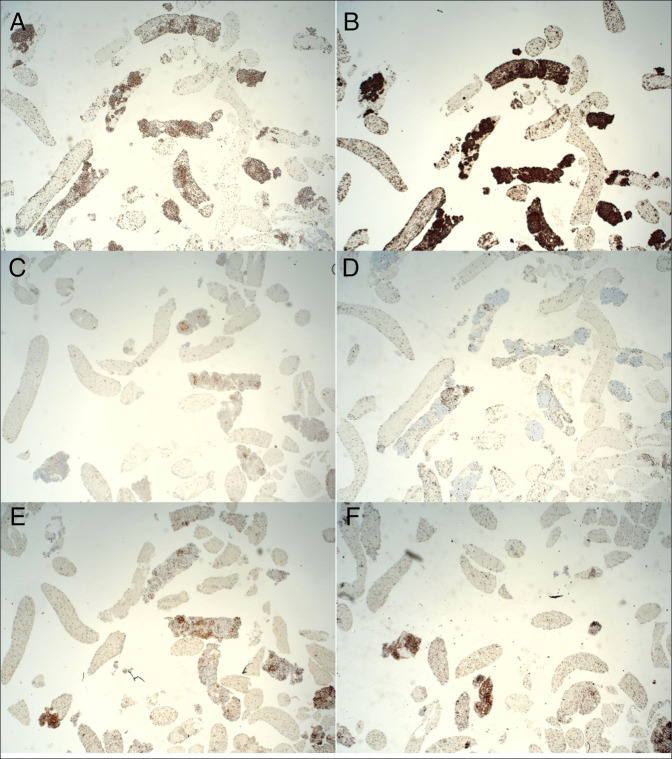
Histologic slides of renal mass biopsy. The image shows (A) aberrant p53 expression, (B) CD20-positive B cells, (C) negative staining for CD10, (D) Ki-67 highlighting 30-40% of neoplastic cells, (E) scattered background T cells on CD3 stain, and (F) scattered background T cells on CD5 stain. All images at 4× magnification.

Pathology of the renal mass revealed mature B-cell lymphoma consistent with low-grade MALT lymphoma with aberrant p53 expression in greater than 80% of neoplastic cells (Figure 3). Morphologic analysis revealed nodular aggregates of small lymphocytes with mature cytologic features. Immunohistochemistry showed that the atypical lymphocytes were CD20-positive B cells, coexpressing CD43, BCL-2, and BCL-6. Neoplastic cells were negative for CD10 and CD23. Kappa and lambda light chains were polytypic. CD3 and CD5 showed scattered background T cells. Additional staining with synaptophysin, chromogranin, CK7, p40, and CDX2 was negative. Ki-67 highlighted 30%-40% of neoplastic cells. Intestinal epithelium showed no evidence of malignancy. The patient has started treatment with bendamustine/rituximab, while also being managed for his rectal adenocarcinoma.

## DISCUSSION

MALT lymphoma is of gastric origin in up to 80% of cases and is strongly associated with *Helicobacter pylori* infection.^1–3^ Duodenal origin is less common,^4^ and renal involvement is exceedingly rare as lymphoid tissue is not natively found in the kidney and typically occurs only in settings of chronic inflammation, such as autoimmune states.^5,7^ Non-Hodgkin lymphoma is a known complication of SLE, and MALT lymphomas have occasionally been described in association with autoimmune diseases, such as Sjogren syndrome or Hashimoto's thyroiditis. There has been only one other reported case of renal MALT lymphoma in a patient with SLE, hypothesized to be from uncontrolled lymphocyte activity causing malignant transformation.^7^

Traditional methods of renal sampling consist of ultrasound or CT-guided percutaneous biopsy. Ultrasound is most common because of its simplicity and efficiency, whereas CT is noted to provide improved visualization of smaller renal masses.^6^ However, both can be limited by size, cystic features, long skin-to-mass distances, upper pole location with hilar involvement, or large body habitus.^8–10^ EUS-guided techniques using fine needle aspiration (FNA) or FNB offer high resolution imaging and more precise targeting of renal masses with otherwise difficult features or positioning.^11^ In addition, EUS-guided procedures can sample multiple intra-abdominal and intraluminal areas under a single procedure.^11^

The feasibility and efficacy of EUS-guided FNA approaches have been well demonstrated^11–14^ with high sensitivity and specificity for cytological diagnosis but fewer cases describe the use of FNB.^15^ FNA uses a thin needle to obtain cells without preservation of histological architecture, whereas FNB samples the tissue itself allowing for histological preservation and subsequently greater diagnostic capabilities.^16^ Risks of EUS sampling are small and include bleeding, infection, and perforation. Most procedures can be performed with monitored anesthesia care and do not necessarily require general anesthesia. EUS-guided and percutaneous biopsies show a similar safety profile, but EUS confers added benefits of minimized patient discomfort and use of smaller needles with Doppler to minimize bleeding risk. Further studies are needed to evaluate exact safety profiles.

An EUS-guided approach was chosen for this case to obtain a diagnostic tissue sample intraluminally, especially in the setting of the patient's prior duodenal lymphoma, in addition to transluminal biopsies, if necessary, all in the same procedure. EUS-guided approaches may be better suited for renal sampling in patients with indications for endoscopy to avoid multiple procedures. The application of EUS-guided renal sampling has also extended beyond renal masses into diagnosis of renal conditions, such as minimal change disease and IgA nephropathy.^17,18^ This supports EUS-guided sampling as a safe and minimally invasive approach to the upper pole or anterior areas that may otherwise be difficult to access.^16^

This case illustrates an exceedingly rare case of renal MALT lymphoma and demonstrates the value of EUS-guided FNB as a minimally invasive approach to histopathologic evaluation of renal masses. With respect to renal cancers, therapeutic endoscopists must work alongside medical oncology, surgical oncology, nephrology, and interventional radiology to help guide best sampling techniques for each patient. This case acts as a building block for further research in the developing field, Endonephrology.

## DISCLOSURES

Author contributions: E. Holzwanger performed the procedure. E. Tsuchiyose drafted the manuscript. M. Talanian and E. Holzwanger revised and reviewed the manuscript. E. Holzwanger is guarantor of the article.

Financial disclosure: None to report. Conflict of Interest: Erik Holzwanger is a medical consultant for Boston Scientific. The remainder of authors have no conflicts of interest.

Informed consent was obtained for this case report.

## ABBREVIATIONS:

CT: computed tomography
EUS: endoscopic ultrasound
FNA: fine needle aspiration
FNB: fine needle biopsy
MALT: mucosa-associated lymphoid tissue
SLE: systemic lupus erythematosus

## References

[1] Xu X Wang Z Yu Y . Evaluation of the clinical characteristics and prognostic factors of gastrointestinal mucosa-associated lymphoid tissue (MALT) lymphoma. J Gastroenterol Hepatol 2014;29(9):1678–84. 24730769 10.1111/jgh.12615

[2] Raderer M Kiesewetter B Ferreri AJM. Clinicopathologic characteristics and treatment of marginal zone lymphoma of mucosa-associated lymphoid tissue (MALT lymphoma). CA Cancer J Clin 2016;66(2):153–71. 26773441 10.3322/caac.21330

[3] Isaacson PG. Mucosa-associated lymphoid tissue lymphoma. Semin Hematol 1999;36(2):139–47. 10319382

[4] Na HK Won SH Ahn JY . Clinical course of duodenal mucosa-associated lymphoid tissue lymphoma: Comparison with gastric mucosa-associated lymphoid tissue lymphoma. J Gastroenterol Hepatol 2021;36(2):406–12. 32573049 10.1111/jgh.15157

[5] Makino T Miwa S Koshida K Kawashima A. Mucosa-associated lymphoid tissue lymphoma involving the kidney: A case report and review of the literature. Int Cancer Conf J 2015;5(2):82–9. 31149432 10.1007/s13691-015-0234-6 PMC6498318

[6] Marcelin C Ambrosetti D Bernhard JC Roy C Grenier N Cornelis FH. Percutaneous image-guided biopsies of small renal tumors: Current practice and perspectives. Diagn Interv Imaging 2017;98(9):589–99. 28844612 10.1016/j.diii.2017.07.008

[7] Mortlock AM Lim CS Morgan H . Renal MALToma: An unusual lymphoma in a patient with lupus. Lupus 2006;15(9):613–5. 17080919 10.1177/0961203306071920

[8] Prince J Bultman E Hinshaw L . Patient and tumor characteristics can predict nondiagnostic renal mass biopsy findings. J Urol 2015;193(6):1899–904. 25498574 10.1016/j.juro.2014.12.021 PMC4573549

[9] Park SY Park BK Kim CK Kwon GY. Ultrasound-guided core biopsy of small renal masses: Diagnostic rate and limitations. J Vasc Interv Radiol 2013;24(1):90–6. 23206333 10.1016/j.jvir.2012.09.007

[10] Lawrence EM Lubner MG Pickhardt PJ Hartung MP. Ultrasound-guided biopsy of challenging abdominopelvic targets. Abdom Radiol (NY) 2022;47(8):2567–83. 34322727 10.1007/s00261-021-03223-4

[11] DeWitt J Gress FG Levy MJ . EUS-guided FNA aspiration of kidney masses: A multicenter U.S. experience. Gastrointest Endosc 2009;70(3):573–8. 19560139 10.1016/j.gie.2009.04.006

[12] Lopes RI Moura RN Artifon E. Endoscopic ultrasound-guided fine-needle aspiration for the diagnosis of kidney lesions: A review. World J Gastrointest Endosc 2015;7(3):253–7. 25789096 10.4253/wjge.v7.i3.253 PMC4360444

[13] Moura DTH McCarty TR Jirapinyo P . Endoscopic ultrasound fine needle aspiration vs fine needle biopsy in solid lesions: A multi-center analysis. World J Clin Cases 2021;9(34):10507–17. 35004982 10.12998/wjcc.v9.i34.10507 PMC8686153

[14] Law R Wobker S Grimm IS Baron TH. Endoscopic ultrasonography-guided fine needle aspiration of kidney masses. Gastroenterology 2015;148(7):1282–3. 25770084 10.1053/j.gastro.2015.03.008

[15] Chang KJ Katz KD Durbin TE . Endoscopic ultrasound-guided fine-needle aspiration. Gastrointest Endosc 1994;40(6):694–9. 7859967

[16] Kwong J May G Ordon M. Endoscopic ultrasound-guided trans-duodenal fine-needle biopsy of a small renal mass: Case report and review of the literature. Afr J Urol 2021;27(1):148.

[17] Rathod V Kulkarni A Sahu M . Endoscopic ultrasound-guided renal parenchymal biopsy: A series of two cases. J Surg 2023;8:1937.

[18] Sundaram S Patil P Jain A . Endoscopic ultrasound-guided renal biopsy: An alternative to transjugular renal biopsy in high-risk patients. Endoscopy 2022;54(6):E285–6. 34215009 10.1055/a-1519-6365

